# Effects of phenolic constituents of daylily flowers on corticosterone- and glutamate-treated PC12 cells

**DOI:** 10.1186/s12906-017-1582-x

**Published:** 2017-01-21

**Authors:** Huan Tian, Fei-Fei Yang, Chun-Yu Liu, Xin-Min Liu, Rui-Le Pan, Qi Chang, Ze-Sheng Zhang, Yong-Hong Liao

**Affiliations:** 1 Institute of Medicinal Plant Development, Chinese Academy of Medical Sciences and Peking Union Medical College, 151 Malianwa North Road, Haidian District, Beijing, 100193 People’s Republic of China; 2 Key Laboratory of Food Nutrition and Safety, Ministry of Education, College of Food Engineering and Biotechnology, Tianjin University of Science & Technology, Tianjin, 300457 People’s Republic of China; 3 National Key Laboratory of Human Factors Engineering, China Astronaut Research and Training Centre, Beijing, 100094 People’s Republic of China

**Keywords:** Hemerocallis citrina, PC12 cells, Phenolic acid derivatives, Flavonoids, Neuroprotection, Neurotransmitters

## Abstract

**Background:**

Daylily flowers, the flower and bud parts of *Hemerocallis citrina* or *H. fulva*, are well known as Wang-You-Cao in Chinese, meaning forget-one’s sadness plant. However, the major types of active constituents responsible for the neurological effects remain unclear. This study was to examine the protective effects of hydroalcoholic extract and fractions and to identify the active fractions.

**Methods:**

The extract of daylily flowers was separated with AB-8 resin into different fractions containing non-phenolic compounds, phenolic acid derivatives and flavonoids as determined using UPLC-DAD chromatograms. The neuroprotective activity was measured by evaluating the cell viability and lactate dehydrogenase release using PC12 cell damage models induced by corticosterone and glutamate. The neurological mechanisms were explored by determining their effect on the levels of dopamine (DA), 5-hydroxy tryptamine (5-HT), γ-aminobutyric acid (GABA), noradrenaline (NE) and acetylcholine (ACh) in the cell culture medium measured using an LC-MS/MS method.

**Results:**

Pretreatment of PC12 cells with the extract and phenolic fractions of daylily flowers at concentrations ranging from 0.63 to 5 mg raw material/mL significantly reversed corticosterone- and glutamate-induced neurotoxicity in a dose-dependent manner. The fractions containing phenolic acid derivatives (0.59% w/w in the flowers) and/or flavonoids (0.60% w/w) exerted similar dose-dependent neuroprotective effect whereas the fractions with non-phenolic compounds exhibited no activity. The presence of phenolic acid derivatives in the corticosterone- and glutamate-treated PC12 cells elevated the DA level in the cell culture medium whereas flavonoids resulted in increased ACH and 5-HT levels.

**Conclusion:**

Phenolic acid derivatives and flavonoids were likely the active constituents of daylily flowers and they conferred a similar extent of neuroprotection, but affected the release of neurotransmitters in a different manner.

## Highlights

The hydroalcoholic extract was separated into phenolic and non-phenolic factions.

Phenolic fractions contained phenolic acid derivatives and flavonoids.

Phenolics but not non-phenolics showed neuroprotection to PC12 damaged cells.

Phenolic acid derivatives affected the release of dopamine and noradrenaline.

Flavonoids affected the release of serotonin noradrenaline and acetylcholine.

## Background

Daylily flowers, commonly called yellow flower vegetable (Huang-Hua-Cai), golden needle vegetable (Jin-Zhen-Cai) and Xuan-Cao flower in China, are the flower and bud parts of *Hemerocallis citrina* Baroni or *H. fulva* (L.) L.. Traditionally, apart from being food materials, the herb has been used as an anodyne, febrifuge, sleep-promoting and sedative agent, and for stimulating the secretion of milk for women in childbirth in China [1]. It is also well known as Wang-You-Cao in Chinese, meaning forget-one’s sadness plant, and has been recorded to exhibit antidepressant effects in ancient medical books of China, including the famous textbook “Compendium of Materia Medica”.

In modern pharmacological and clinical studies, the neurological actions of daylily flowers have been documented. Several studies showed that significant sleep-promoting and sedative effects were elicited in mice treated with daylily flowers or the extracts compared with mice in control group [2–4]. In a clinical study, daylily flowers exhibited significant better efficacy in treating insomnia than the control treatment with diazepam [2]. In addition, many previous studies have also demonstrated that the hydroalcoholic extracts of daylily flowers conferred antidepressant-like effects in acute stress-induced, chronic unpredictable mild stress-induced and corticosterone-induced depression-like models of rodents and improving learning and memory in animal models [5–10]. In these studies, the underlying mechanisms for the central nervous system (CNS) modulating effects were not entirely clear and were considered to at least partly involve in the anti-inflammatory property and the ability to mediate the brain levels of monoamine neurotransmitters such as serotonin (5-HT), noradrenaline (NE) and dopamine (DA) [7, 9, 10].

The major constituents responsible for the CNS modulating effects remain unclear. Some previous studies speculated that such effects might be related to flavonoids [8, 9]. Indeed, Du and colleagues found that in comparison to the aqueous extract, the higher flavonoid content in the alcohol extracts of daylily flowers resulted in better biological activities [9]. In particular, the work also revealed that the combination of rutin and hesperidin, two main flavonoid ingredients, displayed similar antidepressant-like effect to the alcohol extracts. In our previous in vivo screening study on the active ingredients of daylily flowers, the fraction containing ~60% flavonoids exhibited significant antidepressant-like effects whereas the fraction lack of flavonoids showed no effect in mice model by forced swimming test, tail suspension test and antagonism reserpine experiments [11]. However, daylily flowers contain various types of chemical ingredients including flavonoids, phenolic acids and derivatives, di- and tri-terpenes, essential oils, steroidal saponins, alkaloids, amino acids, polysaccharides and so on [12], the potential neurological effects of other constituents in daylily flowers are largely uninvestigated. Therefore, the objectives of this study were to separate the extracts of daylily flowers into different fractions and to evaluate the neuroprotective effects of each fraction using PC12 cell damage models induced by corticosterone and glutamate with also a view to identifying the active fractions and their potential effect on the neurotransmitter release in the cell culture medium.

## Methods

### Plant materials and reagents

*Hemerocallis citrina* was obtained from Qidong county, Hunan province, China. The rat pheochromocytoma cells (PC12) were purchased from the Cell Culture Center of Chinese Academy of Medical Sciences (Beijing, China). Dulbecco’s modified eagle medium (DMEM), penicillin, streptomycin, nonessential amino acids, and fetal bovine serum were purchased from Gibco (New York, USA). MTT (3-(4,5-dimethylthiazol-2-yl)-2,5-diphenyltetrazolium bromide), corticosterone and hydrogen peroxide, DA, 5-HT, γ-aminobutyric acid (GABA), NE, acetylcholine (ACh) and 3, 4-dihydroxy benzylamine (DHBA) were purchased from Sigma-Aldrich (St. Louis, MO, USA). Glutamate was purchased from Amresco (Solon, USA). Lactate dehydrogenase (LDH) diagnostic kit was purchased from Nanjing Jiancheng Bioengineering Institute (Nanjing, China). Acetonitrile and MeOH of HPLC grade were obtained from Merck (Darmstadt, Germany), and formic acid of HPLC grade was from Dima (Lake Forest, CA, USA). Water was purified by a Milli-Q water purification system (Millipore, MA, USA). All other chemicals and reagents were of analytical grade.

### Ultra Performance Liquid Chromatography (UPLC) chromatographic and UPLC/MS conditions

The UPLC system consisted of a Waters Acquity Ultra Performance LC System, a Waters photodiode array detector, Empower software (Waters, Milford, MA, USA), and a Kinetex C18 column (100 mm × 2.1 mm, 1.7 μm, Phenomenx, USA). The column temperature was set at 30 °C. A gradient mobile phase system consisting of acetonitrile:H_2_O (3:97 to 20:80 from 0 to 20 min, 20:80 to 80:20 from 20 to 22 min, 80:20 to 3:97 from 22 to 23 min, 3:97 from 23 to 27 min) with 0.1% formic acid as a modifier was used to elute the analytes after 2 μL of sample solution was applied onto the column whereas the detection wavelength was set at 340 nm [12]. The flow rate was 0.2 mL/min and the sample temperature was maintained at 4 °C.

For the identification of chemical constituents, UPLC/MS analysis was performed using a Waters ACQUITY UPLC system (Waters Corporation, Milford, MA, USA) coupled with a Waters Micromass Q-TOF micro™ Synapt High-Definition Mass Spectrometer (Manchester, UK) equipped with electrospray ionization source (ESI) operating in positive ion mode. The main parameters such as capillary voltages, sample cone voltage and extraction cone voltage were set at 3.0 kV, 40 V and 4.0 V, respectively. Nitrogen was used as the drying gas. The desolvation gas rate was set to 800 L/h at 450 °C, the cone gas rate at 40 L/h, and the source temperature at 120 °C. The scan time and inter scan delay were set 0.15 and 0.02 s, respectively.

### Extraction and separation

The plants came from Qidong (Hunan, China) and were authenticated by Professor Ben-Gang Zhang, the Institute of Medicinal Plant Development (IMPLAD), Chinese Academy of Medical Sciences and Peking Union Medical College, Beijing, China. And the voucher specimen (No. 20150507) was deposited in the herbarium of IMPLAD. Briefly, the raw material of *H. citrina* flowers (250 g) was extracted once with 2.5 L of 90% EtOH under reflux using a water bath for 2 h and then extracted twice with 1.5 L of 70% EtOH under reflux using a water bath for 1 h. The extract solutions were combined, filtered and subsequently concentrated by using a rotary evaporator under reduced pressure at 60 °C to completely remove the ethanol. The concentrated crude extract was diluted to 500 mL and applied to AB-8 macroporous adsorption resin column chromatography (1000 g), followed by eluting with 4 L water, 5 L 10% EtOH, 5 L 30% EtOH, 5 L 50% EtOH and 5 L 95% EtOH, the corresponding elutes were concentrated under reduced pressure at 60 °C and lyophilized into powders [13]. The 10%, 30% and 50% EtOH fractions were further subjected to separation in Sephadex LH20 and each fraction was divided into two fractions, namely phenolic and non- phenolic parts, respectively.

### Cell culture and treatment

PC12 cells were cultured in DMEM supplemented with 10% heat-inactivated fetal bovine serum, 5% heat-inactivated horse serum, 100 U/mL penicillin and 100 μg/mL streptomycin in a humidified 95 air and 5% CO_2_ atmosphere at 37 °C. For all experiments, cells in the logarithmic phase were harvested. To study the protective effect of *H. citrina* extract and fractions, the PC12 cell damage models were induced by corticosterone (250 μmol/L) and glutamic acid (20 mmol/L). PC12 cells were divided into non-treated control, damage model and damage models administrated with test extracts or fractions in all experiments. The PC12 cells were cultured in a 96-well plate at a density of 1 × 10^4^ cells/well. Then cells were treated with corticosterone or glutamate and incubated for 24 h according to previous studies [14, 15], DMEM with different concentrations (equivalent to 0.3125, 0.625, 1.25, 2.5 and 5.0 mg raw material/mL) of extracts or fractions were added and incubated for 24 h.

### MTT and LDH activity assays

The Cell viability was determined using an MTT assay. After incubation, the PC12 cells were treated with MTT solution (working concentration at 0.5 mg/mL) for 4 h at 37 °C. Subsequently, the formazan crystals formed in the intact cells were solubilized with 150 μl of DMSO, and absorbance at 470/590 nm was measured with a microplate reader (Spectrafluor, TECAN, Sunrise, Austria). Cell viability was expressed as a percentage of the control.

The cell damage was evaluated by determining the released LDH using a LDH diagnostic kit according to the manufacturer’s protocol. At the end of incubation, the medium was collected, 100 μL of the medium was added to a polystyrene cuvette containing 1 mL of LDH reagent. The cuvette was immediately placed into a spectrophotometer and maintained at 37 °C. After 1 min-stabilization, the absorbance at 340 nm was recorded at 1 min intervals for 3 min. The change in absorbance was expressed in concentration units per liter. All experiments were carried out in triplicate.

### Measurement of neurotransmitter levels in PC12 cell culture medium

The determination of neurotransmitter levels was used a previously reported LC-MS/MS method [16]. The PC12 cells were cultured in 24-well plate with a density of 5 × 10^4^ cells/well following the protocol described in the Section 2.4. After treatment and incubation, the culture medium was harvested whereas the cells were detached from the wells by trypsin treatment and pelleted cells were then lysed by sonication in 0.1 M perchlorate and centrifugated at 12,000 rpm for 3 min, and supernatants were used for the determination of total protein content. The total protein contents of cell supernatants were measured using BCA protein assay following the manufacture’s protocol. Prior to LC-MS/MS analysis, 20 μL of 30 μg/mL DHBA (internal standard) was added to the culture medium, which was subjected to protein precipitation by adding MeOH at a volume ratio of MeOH:H_2_O 2:1. The samples were then vortexed and centrifuged, and the supernatant were subjected to freeze-drying. The freeze-dried samples were reconstituted in 200 μL MeOH:H_2_O (1:1) and centrifugated at 12,000 rpm for 3 min. The neurotransmitter levels in the medium were normalized by the corresponding total protein content. Two independent experiments were performed with a representative experiment shown.

### Statistical analysis

The statistical analyses were performed with SPSS statistics 17.0. All values were expressed as mean ± SD, unless stated. Data were analyzed using the two tailed, unpaired Student’s *t*-test or factorial analysis of variance (ANOVA), followed by post hoc comparisons using the Dunnett’s or Student-Newman-Keuls (SNK) tests if the ANOVA manifested a significant difference. A two-tailed *P* value of 0.05 or less was taken to indicate statistical significance.

## Results

### Extraction and separation

**Fig. 1:**
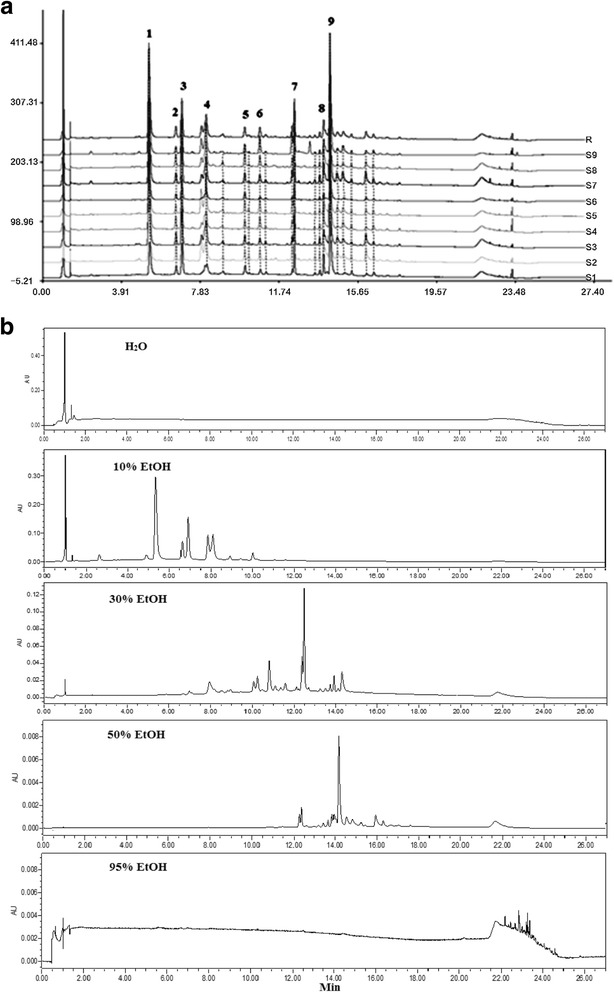
Chromatographic fingerprints of nine batches of *Hemerocallis citrina* samples (**a**) and the typical UPLC chromatograms of *Hemerocallis citrina* fractions eluted with water (with the yield (w/w) of total extract at 21.7%, and yield of water fraction at 16.9%), 10% (the yields of EtOH fraction, Phenolic part and non-phenolic part were 1.20%, 0.59% and 0.61%, respectively), 30% (the yields of EtOH fraction, Phenolic part and non-phenolic part were 0.62%, 0.44% and 0.18%, respectively), 50% (the yields of EtOH fraction, Phenolic part and non-phenolic part were 0.80%, 0.60% containing 0.30% of rutin and 0.20%, respectively) and 95% EtOH (with the yield ratio at 1.54%) sequentially on an AB-8 macroporous resin column (**b**)

The UPLC chromatographic fingerprint of phenolic compounds was used for the quality control of *H. citrina* samples in this study. Among the peaks in the UPLC chromatograms from nine batches of herbal samples (Fig. 1a), 9 major compounds were selected for the discrimination study. The crude EtOH extracts of *H. citrina* was further separated using AB-8 macroporous adsorption resin column chromatography and eluted with water, 10%, 30%, 50% and 95% EtOH sequentially to give water, 10%, 30%, 50% and 95% EtOH fractions, respectively. Each fraction was analyzed by UPLC-UV method and the typical chromatograms are shown in Fig. 1b, which shows that the phenolic compounds were present in the 10%, 30% and 50% EtOH fractions and the water and 95% EtOH fractions contained negligible amount of phenolics.

The phenolic constituents of daylily flowers have been previously studied with phenolic acid derivatives and flavonoid glycosides representing the major types of phenolic compounds and naphthalene and xanthone glycosides as the minor parts [1]. In this study, the major constituents were identified using TOF- accurate mass measurement and UV spectra in the UPLC-DAD chromatograms. Based on the high resolution MS, which exhibited dominant ions at *m/z* 361.0891 [M + Na]^+^ and 147.0435 [M + H − quinic acid]^+^ and MS^2^ of *m/z* 361 yielded an abundant ion at *m/z* 147.0435 and a small ion at *m/z* 119.0475 [M + H - quinic acid − CO]^+^, three *para*-coumaroylquinic acids were identified as 3-*para*-coumaroylquinic acid (Peak 2), 4-*para*-coumaroylquinic acid (Peak 3), and 5-*para*-coumaroylquinic acid (Peak 4), respectively [17]. The peak 1 was identified as chlorogenic acid owing to the MS, which gave two abundant ions at *m/z* 377.0830 [M + Na]^+^ and 163.0391 [M + H − quinic acid]^+^ [17], whereas Peak 9 was predicted to be rutin based on the MS spectra that exhibited abundant ions at *m/z* 633.1436 [M + Na]^+^, 465.1063 [M + H − rhamnose]^+^and 303.0502 [M + H − rhamnose − glucose]^+^ [18]. Peaks 5–8 were unknown, however, Peak 6 was predicted to be a phenolic acid derivatives and peak 5, 7 and 8 were unknown flavonoids based on the UV spectra. The chromatographic data suggested that the 10% EtOH fraction mainly consisted of phenolic acid derivatives whereas the 50% EtOH fraction contained mostly flavonoids.

Considering that the 10%, 30% and 50% EtOH fractions contained both phenolic and non-phenolic compounds, a further separation was carried out using a Sephadex LH-20 column, where phenolic compounds displayed longer retention time than the non-phenolic constituents. The separation of phenolics from the non-phenolic compounds was confirmed in UPLC chromatograms (Data not shown).

### Effect on corticosterone-induced cytotoxicity of PC12 cells

**Fig. 2:**
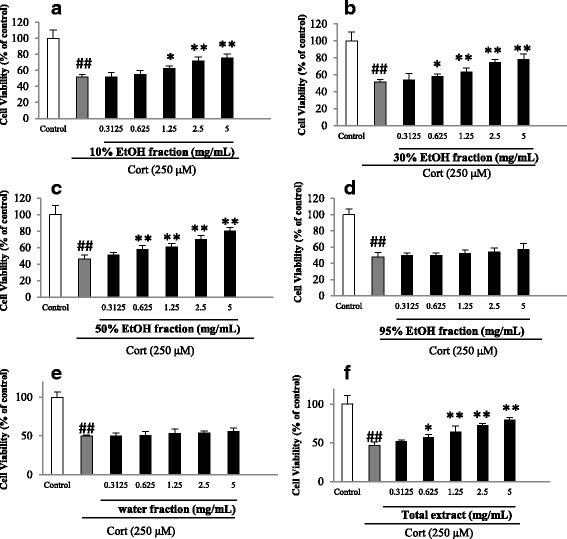
Effects of *Hemerocallis citrina* extract and fractions (**a**-**f**) on corticosterone (Cort) -induced cytotoxicity of PC12 cells determined by MTT assay (mean ± SD, *n* = 5). ##*P* < 0.01 as compared with control group; *P < 0.05 and **P < 0.01 as compared with the corticosterone group

**Fig. 3:**
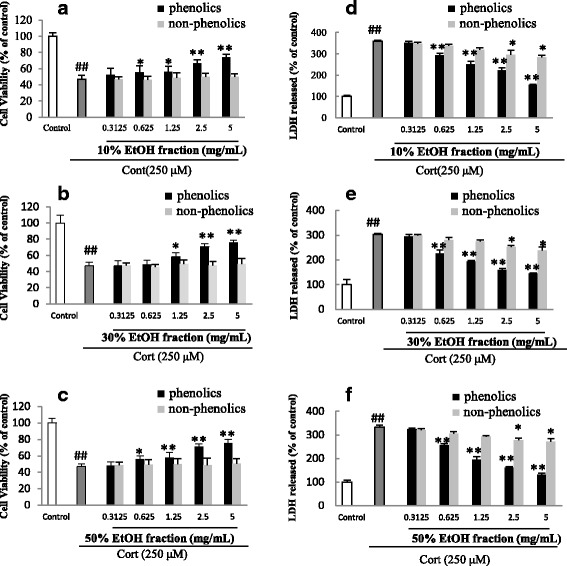
Effects of phenolic and non-phenolic parts (**a**-**f**) of *Hemerocallis citrina* fractions on the cell viability and LDH leakage of corticosterone (Cort) -treated PC12 cells (Mean ± SD, *n* =6). ##*P* < 0.01 as compared with control group; *P < 0.05 and**P < 0.01 as compared with the corticosterone group

The viability of PC12 cells exposed to corticosterone for 24 h significantly decreased relative to the control group (*P* < 0.01) with the survival rates varying from 46 to 51% of the control when measured by an MTT assay (Figs. 2 and 3). When cells were pretreated with the extract of *H. citrina*, the viability of corticosterone treated PC12 cells appeared to increase in a dose-dependent manner. In this study, the test concentrations of extract or fractions were set between 0.31 and 5.0 mg raw herb/mL. The survival rates significantly increased compared to the corticosterone group (*P* < 0.05 or 0.01) when the dose of the extract was equivalent to 0.625 mg/mL of raw herb or higher (Fig. 2). In addition, the eluted fractions from AB-8 column were found to exhibit different effect on the corticosterone-induced cytotoxicity of PC12 cells. The 10%, 30% and 50% EtOH fractions, which contained phenolic compounds, appeared to exert a dose-dependent protecting effect on the cytotoxicity of PC12 cells similar to the total extract whereas no protective ability was shown in the water or 95% EtOH fractions (Fig. 2).

Subsequently, the protective effects of phenolic and non-phenolic constituents from the 10%, 30% and 50% EtOH fractions on the viability of corticosterone treated PC12 cells were evaluated using both MTT assay and LDH leakage (Fig. 3). The results of the MTT assay demonstrated that phenolic constituents from all the three fractions exhibited protective effect in a dose-dependent manner whereas no significant protection was observed in the groups treated with non-phenolic constituents. The data of LDH release, which measures cell lysis for evaluating cell damage, also showed a similar finding that phenolic constituents were responsible for the neuroprotective effects conferred by the 10%, 30% and 50% EtOH fractions (Fig. 3).

### Effects on glutamate-induced cytotoxicity of PC12 cells

**Fig. 4:**
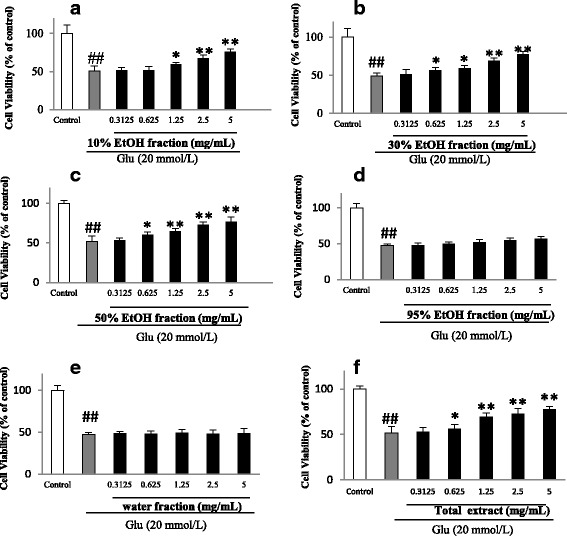
Effects of *Hemerocallis citrina* extract and fractions (**a**-**f**) on glutamate (Glu) -induced cytotoxicity of PC12 cells determined by MTT assay (mean ± SD, *n* = 5). ##*P* < 0.01 as compared with control group; *P < 0.05 and **P < 0.01 as compared with the corticosterone group

**Fig. 5:**
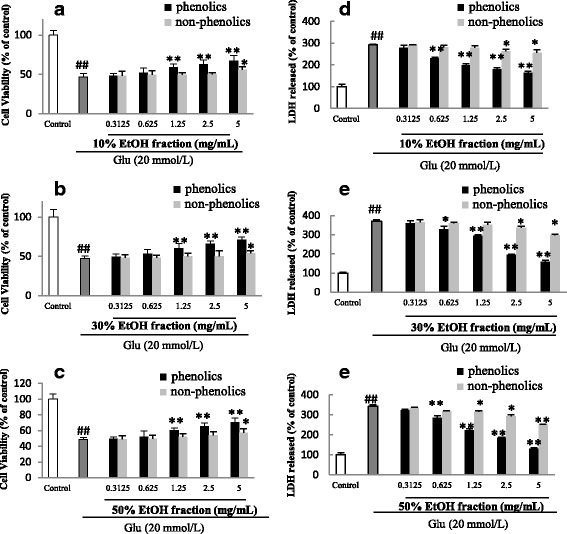
Effects of phenolic and non-phenolic parts (**a**-**f**) of *Hemerocallis citrina* fractions on the cell viability and LDH leakage of glutamate (Glu) -treated PC12 cells (Mean ± SD, *n* = 6). ##*P* < 0.01 as compared with control group; *P < 0.05 and **P < 0.01 as compared with the corticosterone group

Treating PC12 cells with glutamate caused a significant decrease in the cell viability and the survival rates were found between 43 and 51% of the control when measured with MTT assay (Figs. 4 and 5). The results of the MTT assay showed that the total extract, 10%, 30% and 50% EtOH fractions were dose-dependently effective in increasing the viability of glutamate-treated PC12 cells whereas water and 95% EtOH fractions displayed no activity (Fig. 4). In addition, the data from both MTT assay and LDH release experiments in Table 1 supported that the protective effects conferred by 10%, 30% and 50% EtOH fractions were attributable to their phenolic constituents rather than the non-phenolic constituents.

**Table. 1:** Effects of *Hemerocallis citrina* fractions (HF) on the extracellular levels (ng/mg proteins) of dopamine (DA), 5-hydroxy tryptamine (5-HT), γ-aminobutyric acid (GABA), noradrenaline (NE) and acetylcholine (ACh) in corticosterone (Cort) or glutamate (Glu)-treated PC12 cells (mean ± SD, *n* = 5)

	Analyte	EtOH % extract	Control	Cort + Vehicle	Cort + HF (1.25 mg/mL)	Cort + HF (2.5 mg/mL)	Cort + HF (5 mg/mL)
Cort	DA	10%	117.18 ± 14.18	67.40 ± 11.56^##^	98.01 ± 12.04	101.65 ± 24.17**	100.06 ± 24.60**
		30%	157.50 ± 34.50	81.48 ± 49.68^##^	136.33 ± 42.53	127.50 ± 7.94**	136.12 ± 18.09**
		50%	134.18 ± 39.12	87.23 ± 13.26^##^	103.78 ± 14.86	113.98 ± 25.40	117.15 ± 28.17
		10%	0.88 ± 0.04	0.60 ± 0.03^##^	0.78 ± 0.21*	0.91 ± 0.03**	0.91 ± 0.08**
	NE	30%	1.48 ± 0.07	1.18 ± 0.16^#^	1.54 ± 0.10*	1.58 ± 0.35*	1.68 ± 0.06**
		50%	1.34 ± 0.17	0.83 ± 0.20^##^	1.42 ± 0.26**	1.42 ± 0.27**	1.49 ± 0.17**
		10%	15.53 ± 6.20	7.88 ± 4.26^##^	11.89 ± 3.10	12.88 ± 2.65	13.76 ± 2.45
treated	ACh	30%	20.60 ± 0.70	13.57 ± 3.05^##^	19.23 ± 1.85*	20.02 ± 5.05*	20.42 ± 5.25*
cells	GABA	50%	20.88 ± 4.56	8.35 ± 2.41^##^	18.46 ± 0.67**	19.38 ± 2.97**	21.07 ± 3.39**
		10%	97.59 ± 56.99	39.52 ± 14.56^##^	41.62 ± 16.01	57.18 ± 13.74	58.78 ± 12.29
		30%	153.03 ± 20.91	102.35 ± 12.87^##^	120.32 ± 15.55	125.12 ± 37.04	123.93 ± 33.42
	5-HT	50%	114.70 ± 38.33	55.43 ± 19.63^##^	112.06 ± 16.21*	113.40 ± 40.61*	111.75 ± 4.24*
		10%	159.67 ± 20.00	107.76 ± 7.93^##^	119.09 ± 14.71	129.33 ± 17.66	121.22 ± 38.25
		30%	195.50 ± 47.78	111.15 ± 31.92^##^	170.26 ± 38.25	204.25 ± 32.39**	205.86 ± 48.56**
	DA	50%	150.03 ± 8.61	93.04 ± 9.03^##^	138.74 ± 31.51*	139.38 ± 31.00*	152.20 ± 24.80**
		10%	29.97 ± 1.06	11.54 ± 2.88^##^	19.68 ± 8.22	24.16 ± 9.74*	26.74 ± 5.63**
		30%	49.40 ± 11.72	18.66 ± 9.04^##^	24.11 ± 13.55	28.6 ± 3.65	29.62 ± 7.75
	NE	50%	50.90 ± 23.14	19.61 ± 8.07^##^	28.40 ± 9.75	29.95 ± 10.18	29.64 ± 7.41
		10%	1.94 ± 0.32	1.53 ± 0.12^#^	2.00 ± 0.23	2.00 ± 0.22*	1.99 ± 0.37*
		30%	2.24 ± 0.10	1.58 ± 0.03^##^	1.91 ± 0.22**	1.98 ± 0.19**	1.99 ± 0.23**
		50%	1.96 ± 0.14	1.50 ± 0.14^##^	1.77 ± 0.19	1.88 ± 0.18*	1.83 ± 0.19*
Glu		10%	36.51 ± 1.49	25.97 ± 2.68^##^	27.23 ± 4.63	28.81 ± 6.76	30.95 ± 2.94
treated	ACh	30%	47.60 ± 19.10	28.45 ± 1.47^#^	36.79 ± 5.23	42.43 ± 3.50*	43.65 ± 3.90*
cells		50%	70.42 ± 13.11	32.36 ± 3.43^##^	51.48 ± 3.93*	53.71 ± 12.87**	57.03 ± 18.05**
		10%	133.73 ± 11.64	51.84 ± 6.36^##^	88.69 ± 12.20**	101.46 ± 19.89**	105.08 ± 16.04**
	GABA	30%	163.25 ± 6.36	74.51 ± 27.86^##^	128.30 ± 44.41*	133.05 ± 20.81*	133.88 ± 28.40*
		50%	198.50 ± 16.74	90.44 ± 25.83^##^	151.50 ± 18.64**	154.75 ± 17.21**	150.94 ± 31.12**
		10%	224.49 ± 24.98	152.40 ± 18.27^##^	177.35 ± 22.54	175.73 ± 36.75	183.31 ± 28.40
	5-HT	30%	211.75 ± 22.14	166.51 ± 24.52^#^	172.98 ± 36.20	205.05 ± 19.46*	207.58 ± 24.62*
		50%	173.50 ± 15.63	107.54 ± 32.38^##^	152.60 ± 20.64**	156.50 ± 23.87**	154.02 ± 9.52**

^##^ *P* < 0.01 as compared with control group; *P < 0.05 and **P < 0.01 as compared with the corticosterone/glutamate group

### Effects on neurotransmitter release in the culture medium of corticosterone-treated PC12 cells

In the medium of corticosterone-treated PC12 cells, the contents of neurotransmitters including DA, NE, Ach, GABA and 5-HT were analyzed in the presence or absence of 10%, 30% and 50% EtOH fractions. As shown in Table 1, the presence of 10 and 30% EtOH fractions significantly increased the levels of DA and NE, and the levels of DA, NE, Ach and 5-HT in the medium of PC12 cells, respectively whereas the treatment with 50% EtOH fraction significantly affected the levels of NE, Ach, GABA and 5-HT, but not DA.

### Effects on neurotransmitter release in the culture medium of glutamate -treated PC12 cells

The effects of phenolic constituents of daylily flowers on neurotransmitter release in the culture medium of glutamate-treated PC12 cells are shown in Table 1, which suggested that the treatment with the 10%, 30% or 50% EtOH fractions significantly enhanced the release of NE and GABA compared with the model group (*P* < 0.05 or 0.01). In addition, the results indicated that the DA release in the medium significantly increased by the presence of 10% EtOH fraction (*P* < 0.05 or 0.01) but not the 30% and 50% EtOH fractions (*P* > 0.05) whereas the treatment with either 30% or 50% EtOH fractions but not 10% EtOH fraction significantly augmented the release of Ach and 5-HT.

## Discussion

Daylily flowers have been ethnopharmacologically used to treat various CNS disorders, such as depression and insomnia and the neuropharmacological actions have also been demonstrated in animal models [1, 4, 7, 9]. However, the major constituents responsible for the CNS modulating effects are yet clear, albeit that the flavonoids are assumed to play a role in the antidepressant-like effects. In this study, the different types of chemical constituents in the hydroalcoholic extract of daylily flowers were fractionated using AB-8 macroporous adsorption resin column. Upon analyzing with UPLC chromatograms, the phenolic constituents appeared to be mainly present in 10%, 30% and 50% EtOH fractions whereas the water and 95% EtOH fractions consisted of non-phenolic constituents. Further analysis revealed that the 10% EtOH fraction comprised mainly phenolic acids and their esters of quinic acid whereas the 50% EtOH fraction contained mostly flavonoids. The separation of phenolic acid derivatives in the 10% EtOH fraction from flavonoids in the 50% EtOH fraction was confirmed in the UPLC chromatogram (Fig. 1b). The different retention times in the UPLC chromatograms between phenolic acid derivatives and flavonoids from daylily flowers were in well agreement with previous findings [12].

Upon separation of different fractions, the neurological actions of the obtained fractions and parts were assessed using PC12 cell damage models induced by corticosterone and glutamate, two in vitro models extensively adopted for assessing neuroprotection relevant to in vivo antidepressant activity [14, 19]. Rat pheochromocytoma (PC12) is a cell line which has an embryonic origin from the neural crest, and it has been employed as a useful model to elucidate the mechanisms of cerebral disease because of its extreme versatility for pharmacological manipulation, ease of culture, and the large amount of information on their proliferation and differentiation, including releasing neurotransmitters, e.g., dopamine, norepinephrine etc. [20]. Corticosterone, glutamate and oxidative stresses play a critical role in the pathogenic cascade in neurological disorders, and corticosterone, glutamate and H_2_O_2_ induced PC12 cell injury have been widely used as in vitro models for neuroprotection study. For example, it has been shown that corticosterone or glutamate can induce cellular damage in PC12 cells and antidepressants could confer protection to PC12 cells by mitigating the corticosterone/glutamate-induced neurotoxicity [19, 21]. The present results demonstrated that the hydroalcoholic extract of daylily flowers possessed the protective activity against corticosterone- and glutamate-induced damage in PC12 cells and the protective effects were highly related to the presence of phenolic constituents. Indeed, the lack of phenolic constituents in the water and 95% EtOH fractions did not result in any neuroprotective activity at all whereas the presence of phenolic compounds in 10%, 30% and 50% EtOH fraction led to significant neuroprotection in a dose-dependent manner. In addition, both MTT and LDH release data supported that the phenolic, rather than the non-phenolic compounds in 10%, 30% and 50% EtOH fractions exerted the protective effects. Therefore, it could be deduced that the phenolic constituents were responsible for the neuroprotective effect of *H. citrina* extract. When compared the protective ability between the 10% (phenolic acid derivatives) and 50% (flavonoids) EtOH fractions, the MTT and LDH release data showed that there was no significant difference, indicating that both phenolic acid derivatives and flavonoids were active in attenuating the PC12 cell damage induced by either corticosterone or glutamate. The phenolic acid constituents present in daylily flowers are not yet fully characterized but include at least coumaroylquinic acids and caffeoylquinic acid [12]. Caffeoylquinic acids have been previously demonstrated to possess neuroprotective ability [22, 23].

Glutamate exerts both excitotoxic and toxic effects on PC12 cells. The excitotoxicity is due to the over activation of NMDA receptors whereas glutamate-induced cytotoxicity is mediated through the inhibition of cystine uptake leading to depletion of GSH and oxidative toxicity [24]. The mechanisms underlying corticosterone-induced cytotoxicity in PC12 cells were yet elucidated, possibly involving the dysfunctions of different pathways such as glycerophospholipid metabolism, sphingolipid metabolism, oxidation of fatty acids, glycerolipid metabolism and sterol lipid metabolism [25]. Several studies suggested that the neuroprotective effect against corticosterone-induced cytotoxicity in PC12 cells was related to the inhibition of oxidative stress [26]. Botanical phenolics are known to act as antioxidants and therefore it is conceivable that the phenolic fractions of *H. citrina* extract conferred protective effects against corticosterone- and glutamate-induced cytotoxicity in PC12 cells likely via inhibition of oxidative stress.

Deregulation of neurotransmitters such as DA, NE, ACh, GABA and 5-HT is a key feature of CNS disorders and increases in serotonergic, noradrenergic, dopaminergic and/or cholinergic neurotransmission are considered to be relevant to the most current treatments [27–30]. In this study, the effects of the phenolic fractions of *H. citrina* extract on the release of DA, NE, ACh, GABA and 5-HT in the cell culture medium of corticosterone- and glutamate-treated PC12 cells were investigated. PC12 cells have previously been used as a model for neurotransmitter release [20]. For example, Kozminski et al. found that upon exposure to L-DOPA, the amount of released catecholamines increased from PC12 cells [31]. The present results showed that there existed differences in the modulation of neurotransmitter release between phenolic acid derivatives (10% EtOH fraction) and flavonoids (50% EtOH fraction). Phenolic acid derivatives appeared to affect the noradrenergic and dopaminergic neurotransmitter system whereas flavonoids enhanced the release of serotonergic, noradrenergic and cholinergic neurotransmitters. In previous animal experiments, daylily flower extracts were reported to elevate the levels of corresponding neurotransmitters in the brain tissues, conferring the antidepressant effects via mediating serotonergic, noradrenergic and dopaminergic systems [7, 9, 10] and flavonoids were assumed to be responsible. Indeed, rutin and hesperidin, the major flavonoid ingredients of daylily flowers [9], were proven to exert antidepressant-like property via the central serotonergic and noradrenergic systems [32, 33]. However, in this study, the dopamine release was only affected by the presence of phenolic acid derivatives rather than flavonoids. Therefore, the phenolic acid derivatives may represent the active constituents responsible for the mediation of dopaminergic system, whereas flavonoids were likely responsible for the modulation of serotonergic and noradrenergic systems.

## Conclusions

The present study investigated effects of hydroalcoholic extract of daylily on cytoprotection and neurotransmitter release in PC12 cells. Our results demonstrated that the hydroalcoholic extract of daylily possessed neuroprotective effects against corticosterone and glutamate-induced damage in PC12 cells and the phenolic constituents including both phenolic acid derivatives and flavonoids, rather than non-phenolics, were likely the active constituents. In addition, this study is the first to demonstrate that the phenolic acid derivatives in daylily conferred similar neuroprotection to the flavonoids and yet the former modulated the neurotransmitter release distinct from the latter constituents.

## Abbreviations

5-HT: 5-hydroxy tryptamine; serotonin
Ach: Acetylcholine
ANOVA: Analysis of variance
CNS: Central nervous system
DA: Dopamine
DHBA: 3, 4-dihydroxy benzylamine
DMEM: Dulbecco’s modified eagle medium
GABA: γ-aminobutyric acid
LDH: Lactate dehydrogenase
MTT: 3-(4,5-dimethylthiazol-2-yl)-2,5-diphenyltetrazolium bromide
NE: Noradrenaline
PC12 cells: The rat pheochromocytoma cells
SNK: Student-Newman-Keuls
UPLC: Ultra Performance Liquid Chromatography

## References

[1] Lim TK (2014). Edible Medicinal and Non-Medicinal Plants: Volume 8, Flowers.

[2] Wang QCP CY, Shi M, Li YP, Zhu XW, Zhang J, Wang JP, Xu A, Kuang YS, Gu GQ, Hu PF, Zhang ZF, Zhang XM (1993). Clinical and experimental report on the treatment of insomnia with Xuan-Cao flower. Shanghai J Tradit Chin Med.

[3] Fan BW J, Xu SF (1996). Experimental observation of the sedative effect of Xuan-Cao flower on mice. Shanghai J Tradit Chin Med.

[4] Uezu E (1998). Effects of Hemerocallis on sleep in mice. Psychiatry Clin Neurosci.

[5] He YH Z, Yang J, Yang Y, Wang T, Zhou YZ (2008). Experimental study on the antidepressant effects of Hemerocallis citrina. J Ningxia Med.

[6] Shen NL ZW, Li JJ, Zhang G, Li XY, Luo XQ, Wang YF, Yao JT, Jin LH (2011). The effects of daylily on the behaviors and learning memory in the depressed rats. Chin J Behav Med Brain Sci.

[7] Gu L, Liu YJ, Wang YB, Yi LT (2012). Role for monoaminergic systems in the antidepressant-like effect of ethanol extracts from Hemerocallis citrina. J Ethnopharmacol.

[8] Lin SH, Chang HC, Chen PJ, Hsieh CL, Su KP, Sheen LY (2013). The Antidepressant-like Effect of Ethanol Extract of Daylily Flowers (Jin Zhen Hua) in Rats. J Tradit Complement Med.

[9] Du BJT XS, Liu F, Zhang CY, Zhao GH, Ren FZ, Leng XJ (2014). Antidepressant-like effects of the hydroalcoholic extracts of Hemerocallis Citrina and its potential active components. BMC Complement Altern Med.

[10] Liu XL, Luo L, Liu BB, Li J, Geng D, Liu Q, Yi LT (2014). Ethanol extracts from Hemerocallis citrina attenuate the upregulation of proinflammatory cytokines and indoleamine 2,3-dioxygenase in rats. J Ethnopharmacol.

[11] Zhai JLT H, Li MQ, Zhang ZS, Liao YH, Chang Q, Pan RL, Liu XM (2015). Screen of active anti-depression ingredients from daylily. Chin Food Addit.

[12] Lin YL, Lu CK, Huang YJ, Chen HJ (2011). Antioxidative Caffeoylquinic Acids and Flavonoids from Hemerocallis fulva Flowers. J Agr Food Chem.

[13] Xu P, Wang KZ, Lu C, Dong LM, Le Zhai J, Liao YH, Aibai S, Yang Y, Liu XM (2016). Antidepressant-like effects and cognitive enhancement of the total phenols extract of Hemerocallis citrina Baroni in chronic unpredictable mild stress rats and its related mechanism. J Ethnopharmacol.

[14] Yanfen D, Li Jiangxia YC (2013). The Pharmacologic Research Progress of Ginsenoside Rh1. Mod Chin Med.

[15] Y-m L, Shen S-n, F-b X, Chang Q, Liu X-m, Pan R-l (2015). Neuroprotection of Stilbenes from Leaves of Cajanus cajan against Oxidative Damage Induced by Corticosterone and Glutamate in Differentiated PC12 Cells. Chin Herb Med.

[16] Zhang LK LT, Sun L, Ye LH, Xiao BX, Cao FR, Liu XM, Chang Q (2013). Simultaneous Determination of Seven Neurotransmitters in Mice Brain Tissue by LC-MS /MS. Chin J Exp Tradit Med Formulae.

[17] Parveen I, Threadgill MD, Hauck B, Donnison I, Winters A (2011). Isolation, identification and quantitation of hydroxycinnamic acid conjugates, potential platform chemicals, in the leaves and stems of Miscanthus x giganteus using LC-ESI-MSn. Phytochemistry.

[18] Chen Y, Luo J, Zhang Q, Kong L (2016). Identification of active substances for dually modulating the renin-angiotensin system in Bidens pilosa by liquid chromatography-mass spectrometry-based chemometrics. J Funct Foods.

[19] Mao QQ, Zhong XM, Feng CR, Pan AJ, Li ZY, Huang Z (2010). Protective effects of paeoniflorin against glutamate-induced neurotoxicity in PC12 cells via antioxidant mechanisms and Ca(2+) antagonism. Cell Mol Neurobiol.

[20] Westerink RH, Ewing AG (2008). The PC12 cell as model for neurosecretion. Acta Physiol.

[21] Li YFLY, Huang WC, Luo ZP (2003). Cytoprotective effect is one of common action pathways for antidepressants. Acta Pharmacol Sin.

[22] Lee SG, Lee H, Nam TG, Eom SH, Heo HJ, Lee CY, Kim DO (2011). Neuroprotective Effect of Caffeoylquinic Acids from Artemisia princeps Pampanini against Oxidative Stress-Induced Toxicity in PC-12 Cells. J Food Sci.

[23] Mikami Y, Yamazawa T (2015). Chlorogenic acid, a polyphenol in coffee, protects neurons against glutamate neurotoxicity. Life Sci.

[24] Kritis AA, Stamoula EG, Paniskaki KA, Vavilis TD (2015). Researching glutamate - induced cytotoxicity in different cell lines: a comparative/collective analysis/study. Front Cell Neurosci.

[25] Zhang H, Zheng H, Zhao G, Tang C, Lu S, Cheng B, Wu F, Wei J, Liang Y, Ruan J (2016). Metabolomic study of corticosterone-induced cytotoxicity in PC12 cells by ultra performance liquid chromatography-quadrupole/time-of-flight mass spectrometry. Mol Biosyst.

[26] Mao QQ, Huang Z, Ip SP, Xian YF, Che CT (2012). Protective effects of piperine against corticosterone-induced neurotoxicity in PC12 cells. Cell Mol Neurobiol.

[27] Davis S, Heal DJ, Stanford SC (1995). Long-Lasting Effects Of an Acute Stress on the Neurochemistry And Function Of 5-Hydroxytryptaminergic Neurons In the Mouse-Brain. Psychopharmacology.

[28] Krystal JH, Sanacora G, Blumberg H, Anand A, Charney DS, Marek G, Epperson CN, Goddard A, Mason GF (2002). Glutamate and GABA systems as targets for novel antidepressant and mood-stabilizing treatments. Mol Psychiatr.

[29] Brunello N, Mendlewicz J, Kasper S, Leonard B, Montgomery S, Nelson JC, Paykel E, Versiani M, Racagni G (2002). The role of noradrenaline and selective noradrenaline reuptake inhibition in depression. Eur Neuropsychopharm.

[30] Millan MJ (2004). The role of monoamines in the actions of established and “novel” antidepressant agents: a critical review. Eur J Pharmacol.

[31] Kirk D, Kozminski DAG, Viviana D, David S, Andrew G, Ewing (1998). Voltammetric and pharmacological characterization of dopamine release from single exocytotic events at rat phaeochromocytoma (PC12) cells. Anal Chem.

[32] Machado DG, Bettio LEB, Cunha MP, Santos ARS, Pizzolatti MG, Brighente IMC, Rodrigues ALS (2008). Antidepressant-like effect of rutin isolated from the ethanolic extract from Schinus molle L. in mice: Evidence for the involvement of the serotonergic and noradrenergic systems. Eur J Pharmacol.

[33] Souza LC, de Gomes MG, Goes AT, Del Fabbro L, Filho CB, Boeira SP, Jesse CR (2013). Evidence for the involvement of the serotonergic 5-HT(1A) receptors in the antidepressant-like effect caused by hesperidin in mice. Prog Neuropsychopharmacol Biol Psychiatry.

